# AECHL-1, a novel triterpenoid, targets tumor neo-vasculature and impairs the endothelial cell cytoskeleton

**DOI:** 10.1007/s10456-015-9466-5

**Published:** 2015-05-08

**Authors:** Aparajita Dasgupta, Mithila A. Sawant, Manish S. Lavhale, Lakshmi-Surekha Krishnapati, Surendra Ghaskadbi, Sandhya L. Sitasawad

**Affiliations:** National Centre for Cell Science, NCCS Complex, S.P. Pune University, Ganeshkhind, Pune, 411007 Maharashtra India; Developmental Biology Group, MACS-Agharkar Research Institute, G.G. Agarkar Road, Pune, 411004 India; Pharmazz India Private Limited, H-6, Site-C, Surajpur Industrial Area, Greater Noida, 201307 Uttar Pradesh India

**Keywords:** Angiogenesis, AECHL-1, HUVEC, Cytoskeleton, HIF-1α

## Abstract

Tumor angiogenesis is characterized by abnormal vessel morphology leading to erratic and insufficient delivery of chemotherapeutics and oxygen, making the tumor core not only highly hypoxic but also unresponsive toward treatment. Such hypoxic conditions promote tumor aggressiveness, leading to the establishment of metastatic disease. Most anti-angiogenic treatments aim toward the destruction of tumor vasculature, which proves countereffective by further increasing its aggressive nature. Hence, developing drugs which target or regulate these processes might lead to a better delivery of chemotherapeutics resulting in tumor shrinkage. Plant-derived natural compounds having a bioactive ingredient, especially triterpenoids, have been known to possess anticancer properties. AECHL-1, a recently isolated novel triterpenoid with proven anticancer potential, is seemingly noncytotoxic toward HEK 293 and HUVECs. Also, cytotoxicity was absent during in vivo studies involving intraperitoneal injections with 5 µg/kg body weight AECHL-1 on SCID mice. When used at subtoxic doses, it was found to be effective in suppression of neo-vessel formation as demonstrated in the chick chorioallantoic membrane, rat aortic rings, Matrigel plugs and xenograft tumors implanted in SCID mice. Tumor vasculature from AECHL-1-treated mice showed greater mural cell coverage and relatively normalized architecture. Investigations into the molecular mechanisms responsible for these observations revealed an effect on the actin cytoskeleton of stimulated HUVECs as well as the VEGFR2-mediated MAPK pathway. AECHL-1 could effectively distinguish between stimulated and nonstimulated endothelial cells. AECHL-1 could also downregulate HIF-1α expression and VEGF secretion under hypoxic conditions, thus reducing the fears of unnecessarily aggravating tumor metastasis as a result of anti-angiogenic therapy. Results obtained from the aforementioned studies make it clear that though AECHL-1 shows promise in discouraging and pruning neo-vasculature, it may not affect existing vasculature as the doses used for the assays are significantly lower than the ones causing endothelial cell death and has potential to be considered as a candidate for therapeutic drug development.

**Electronic supplementary material:**

The online version of this article (doi:10.1007/s10456-015-9466-5) contains supplementary material, which is available to authorized users.

## Introduction

Tumor angiogenesis involves the development of new blood vessels from preexisting ones, as a response to certain pro-angiogenic signals sent out by masses of neoplastic cells in order to sustain their growth into primary tumors, followed by invasion and metastasis [1–3]. Even though tumor vasculature has been an attractive therapeutic target for combating cancer from the time of its recognition, attempts at targeting it have run into several complexities arising due to increased toxicity, developed resistance and enhanced tumor aggressiveness as a result of the treatments [4–6]. Tumor vasculature has a disregard for any vascular hierarchy, forms arteriovenous shunts [7], is tortuous, leaky and tends to run into dead ends with a host of other structural and functional abnormalities [8–10]. Further insights gained from delving into a deeper understanding reveals a stark difference from preexisting normal vasculature. So, although angiogenesis is a process which is needed by the host for wound healing, embryonic development and development of the corpus luteum, it is now possible to selectively target these differences and create drugs which would satisfactorily counter the tumorigenic state without affecting the functions of the host’s preexisting or normally functioning vasculature. Thus, anti-angiogenic therapies have seen a multifaceted onslaught of attacks including agents that neutralize VEGF (e.g., Avastin, VEGF-TRAP), target VEGFR2 and other receptor tyrosine kinases, or inhibit NF-κB and AKT pathways or even target microtubules [11–13].

In the last decade, many investigators have made efforts to identify molecules capable of interacting with receptors expressed in angiogenic vessels, in an attempt to generate new anti-angiogenic drugs or to obtain ligands for targeted delivery of other drugs to tumors. Several known anti-angiogenic leads have been designed from plant extracts [14–19], and many triterpenoids isolated from plant extracts have shown anticancer activities [20]. Synthetic triterpenoid derivatives, including cyano-3,12-dioxooleana-1,9 (11)-dien-28-oic (CDDO), its methyl ester (CDDO-Me), and imidazolide (CDDO-Im) derivatives, have shown potential as a potent anti-angiogenic agent both in vitro and in vivo through the suppression of NF-κB, STAT signaling, enhancing the transcriptional ability of Smad and also by interfering with Keap1 activity [21]. In human umbilical vein endothelial cells, these compounds can inhibit the activation of the extracellular signal-regulated kinase ERK1/2 pathway after stimulation with VEGF.

We have recently isolated and characterized a novel triterpenoid *Ailanthus excelsa* chloroform extract-1 (AECHL-1) (C29H36O10) from the root bark of *Ailanthus excelsa* RoxB (Tree of heaven), a tree belonging to family Simaroubaceae, widely used in Ayurveda [22, 23] as an anti-asthmatic, antispasmodic, bronchodilator, anti-colic pain and anti-diabetic [24]. The compound possesses anticancer activity against a variety of cancer cell lines of different origin and is less toxic, more selective and more effective in the treatment of cancer in comparison with plant-derived anticancer compound paclitaxel and metal-based compound cisplatin. It is efficacious in inhibiting the proliferation of a broad range of cancer cells as well as solid tumors by upregulation of tumor suppressor proteins and cell cycle arrest along with microtubule disassembly. In this study, we have demonstrated the anti-angiogenic potential of AECHL-1 at subcytotoxic concentrations. AECHL-1 has shown the ability to carry out vascular pruning and inhibit neo-vascularization in vivo, without any significant systemic toxicity. The anti-neo-vasculature effect seems to stem from its interference with the actin cytoskeletal dynamics and inhibition of VEGF-mediated VEGFR2 phosphorylation in activated endothelial cells.

## Materials and methods

### Drugs and cell culture

Human umbilical vein endothelial cells (HUVECs) were purchased from Lonza and were cultured in EGM-2 media (Lonza). Cultures were maintained at 37 °C with 5 % CO_2_ in a humidified incubator. In total, 60–80 % confluent cultures were used for all experiments. HUVECs were used within the first five passages. MCF-7 breast adenocarcinoma cell line was obtained from ATCC. GFR Matrigel and BD BioCoat and BD Matrigel invasion chamber were purchased from Becton and Dickson (BD Biosciences, USA). VEGF, ECGS and heparin were from Sigma. AECHL-1 was extracted, purified and characterized as described earlier [22].

### Cell viability assay

Direct interference between different concentrations of AECHL-1 (0–200 mM) and MTT in a cell-free system was not observed; therefore, MTT assay was used to test cell viability in the current system. HUVECs (5000/well) were cultured in 96-well plates for 24 h and were treated with different concentrations of AECHL-1 (0–30 µM) for 48 h at 37 °C. After incubation, cell culture media was replaced with 100 μl of MTT (0.5 mg/ml) solution and was further incubated at 37 °C for 3–4 h. After 4 h, the MTT solution was discarded and replaced with 100 µl of DMSO and incubated at room temperature for 15 min on a shaker for dissolving the formazan crystals, and the absorbance was measured at 570 nm.

### Cell proliferation assay

Proliferation of HUVEC cells was determined by measuring (^3^H) thymidine incorporation. Briefly, aliquots of complete medium containing 5000 cells were distributed into 96-well tissue culture plates. After 24 h, the media was replaced with various concentrations of AECHL-1 (0–30 µM). Six hours after the treatment, 1 µCi/well (^3^H) thymidine (Board of Radiation and Isotope Technology, Mumbai, India) was added and the cultures were incubated further for 42 h at 37 °C. Cells were rinsed and collected in scintillation mixture, and radioactivity incorporated into the DNA was determined with a liquid scintillation counter (Canberra Packard).

### Apoptosis detection assay

HUVECs (50,000/ml) were treated with various concentrations of AECHL-1 (0–40 mM) for 24 h at 37 °C. Cells were harvested after 24 h, and apoptosis was detected by using Annexin V-FITC apoptosis detection reagents (BD Biosciences, USA) using flow cytometry (FACS CALIBUR–BD Biosciences, USA). The data were analyzed using Cell Quest software for determining the percentage of apoptotic cells.

### In vitro tube formation assay

The ability of endothelial cells to form capillary-like structures/tubes on an extracellular matrix material such as Matrigel is exploited by one of the most important tests in determining the pro-/anti-angiogenic effect of any compound [25, 26]. Matrigel 75 µl was added in 96-well plate and allowed to polymerize at 37 °C for 30 min. HUVECs were seeded at the density of 30,000 cells/well/100µl on top of the polymerized Matrigel layer. The cells were treated with either AECHL-1 (5 µM) or VEGF (10 ng/ml) or their combination. Tube formation was captured at 10× magnification on an inverted microscope (NIKON) after 12 h. Images were analyzed using an ImageJ plugin [27]. Minimum of three fields were analyzed per image.

### Scratch wound assay

HUVECs were seeded in 24-well plates and grown up to nearly 100 % confluency. The cells were scratched with a pipette tip to create wounds. Treatment with VEGF (10 ng/ml) and AECHL-1 (5 µM) and in combination with each other was given in serum-free medium after scratch was made. Randomly chosen fields were photographed at 10× magnification with an inverted microscope, and the images were taken at identical locations at the indicated time points. % cell migration was calculated by comparing final gap width to initial gap width using image pro-plus. VEGF-treated wells at the final time point were normalized to 100 % migration.

### Cell invasion assay

HUVECs were suspended in serum-free culture medium and loaded into Matrigel-coated inserts (BD BioCoat, BD Biosciences, USA) placed in a 24-well plate. The lower chamber thus created was filled with 500 µl 20 % FCS-containing culture medium. For experiments involving conditioned medium, the lower chamber was filled with conditioned medium from MCF-7 cultures grown for 24 h in the presence or absence of Deferoxamine (DFO, Sigma-Aldrich, USA) and AECHL-1. After 16 h, the upper surface of the insert was swabbed with a cotton bud and invasive cells on the lower surface were fixed in 3.7 % PFA. The inserts were then stained using 1 % crystal violet and imaged (10×) using an inverted microscope (Nikon). ImageJ was used for counting the invasive cells.

### Chick chorioallantoic membrane assay (CAM)

Fertilized domestic chick embryos purchased from Venkys India Ltd (Pune) were incubated for 3 days at 37 °C. A small hole was punched at the broad end for convenient embryo placement, and a window was cut open and then sealed with micropore tape. On day 7, filter paper saturated with different concentrations of AECHL-1, PBS and Suramin were placed on the CAM, now exposed through the window. After 48 h, the CAM was dissected out following formalin fixation and photographed using Leica MZ6 stereomicroscope at 1.6× magnification. Image analysis for quantification of tertiary and quaternary vessels was carried out manually.

### Ex vivo rat aortic model assay

Wistar rats (6 weeks in age) were killed by CO_2_ asphyxiation according to the Central Animal Ethical Committee procedures and guidelines (NCCS). Thoracic aorta was carefully dissected, cleaned of peri-adventitial fat, spliced into 1–1.5 mm aortic rings and randomized into Matrigel-coated wells. Rings were overlayed with 100 μl Matrigel and incubated in EGM-2 media. Cultures were maintained at 37 °C under controlled humid atmosphere (5 % CO_2_). The effects of AECHL-1, VEGF and the co-treatment of VEGF (10 ng/ml) and AECHL-1(5 µM) were tested by adding them to culture media at day zero. At different times of culture, rings were photographed under clear field illumination by using an inverted microscope with phase contrast mode (Nikon Corp., Tokyo, Japan).Usually, the time span was 8 days. Images were analyzed using an ImageJ plugin. At the end of the eighth day, the culture media was aspirated and assayed for MMP-2 activity by zymography.

### Enzyme-linked immuno sorbent assay (ELISA) for detection of VEGF

MCF-7 cells were pretreated with AECHL-1 for 2 h and then incubated in the presence or absence of DFO and TNF-α for 6 h. Following termination of experiment, cell culture supernatants were harvested and subjected to Sandwich ELISA using the R&D quantikine ELISA kit for determination of secreted VEGF according to the manufacturer’s instructions.

### ELISA for detection of VEGFR2 phosphorylation

HUVECs grown in 10 mm dishes were preincubated for 4 h in the presence or absence of 5 μM AECHL-1 and stimulated with 10 ng/ml VEGF for 5 min to induce VEGFR2 phosphorylation. Cells were then lysed by sonication in 1× cell lysis buffer (CST) followed by centrifugation. The supernatants were then subjected to ELISA using the CST PathScan Sandwich ELISA kit for determination of phosphorylated and total VEGFR2 according to manufacturer’s instructions. Phosphorylation status of VEGFR2 receptor was expressed as a ratio of pVEGFR2 to total VEGFR2.

### In vivo Matrigel plug assay

Female SCID mice (6–8 weeks) were maintained in accordance with the Central Animal Ethical Committee procedures and guidelines (NCCS). Matrigel plug assay was performed as described previously, with certain modifications [21, 28]. Mice were injected subcutaneously with 500 µl Matrigel containing conditioned media from MCF-7, 100 ng/ml VEGF, 10 units of heparin and 2 ng/ml TNF-α (VTH mixture). The injected Matrigel rapidly formed a single, solid gel plug. The mice were subjected to intraperitoneal (i.p) injection with either PBS (control) or 5 μg AECHL-1 for 7 days following the day of Matrigel injection. After 7 days, the skin of the mice was pulled back to expose the Matrigel plug, which remained intact. The plug was excised and fixed in neutral buffered formalin and embedded in OCT compound (Fisher Health Care, USA). Cryosections were produced using a cryotome (Shandon). Quantification of vessel formation was performed by carrying out immunostaining with an anti-CD31 antibody (1:50, BD Bioscience). Briefly, sections were blocked in 10 % FCS and washed 3× with PBS. Sections were then incubated with CD31-FITC antibody for an hour following which were counterstained with DAPI to visualize the nucleus. Images were taken with an Olympus FluoView confocal microscope at 10× and 60× magnification.

### Western blotting

HUVEC and MCF-7 cultures after being subjected to indicated treatments were scraped and homogenized in RIPA buffer (20 mM Tris–HCl pH 7.5, 120 mM NaCl, 1.0 % Triton X100, 0.1 % SDS, 1 % sodium deoxycholate, 10 % glycerol, 1 mM EDTA and protease inhibitor cocktail, Roche). Proteins were isolated in solubilized form, and concentrations were measured by Bradford assay (Bio-Rad protein assay kit). Solubilized protein (30 µg) was denatured in SDS-PAGE sample buffer (Sigma), resolved in 10 % SDS-PAGE and transferred to PVDF (Millipore, USA) membrane followed by blocking of membrane with 5 % non-fat milk powder (w/v) in TBST (10 mM Tris, 150 mM NaCl, 0.1 % Tween 20). The membranes were incubated with mouse monoclonal anti-VEGF antibody (1:1000; SantaCruz) or anti-WAVE-2, pRac/Cdc42, Rac/Cdc42, pPLC-γ1, ERK1/2 and pERK1/2 antibody (1:1000; Cell Signaling Technology) followed by HRP-conjugated appropriate secondary antibodies and visualized by an enhanced chemiluminescence (Pierce) detection system. Membranes were stripped and reprobed with GAPDH primary antibody (1:10000; Sigma) as a protein loading control.

### MCF-7 xenograft studies

Six-week-old female SCID mice were injected subcutaneously (s.c) into the dorsolateral flank with 5 × 10^6^ MCF-7 cells. When tumor volume reached visible proportions, animals were treated i.p with either PBS or AECHL-1 5 µg/kg body weight of the animal for 10 days. After harvesting tumors, part of the tissue was fixed in neutral buffered formalin and subjected to paraffin embedding, while the rest was either embedded in OCT compound and stored at −80 °C or subjected to Western blotting. Cryosections were used to study vascular architecture through IF imaging using a Zeiss confocal microscope. Vessels positive for CD-31, with and without associated SMA-positive cells, were analyzed on 8- and 30-μm-thick sections. Three random fields were counted in four sections of each tumor specimen, and quantification was carried out using ImageJ.

### Immunofluorescence (IF) by confocal laser scanning microscopy (CFLSM) and immunohistochemistry (IHC)

HUVECs and MCF-7 cells were grown on coverslips and treated with AECHL-1 in combination with VEGF, TNF-α or Deferoxamine. Cells grown till 70 % confluence were fixed with 3.7 % paraformaldehyde for 5 min, followed by permeabilization and blocking in 0.2 % Triton X100 and 5 % milk, respectively. Cells were washed three times with 1x PBS and incubated with primary antibody (HIF-1α, WAVE-2, IQGAP-1) and their respective Alexafluor-conjugated secondary antibodies. Nuclei were labeled using DAPI. The conditions for incubations, concentration of primary and the secondary labeled antibodies were standardized. F-actin was visualized using phalloidin (Molecular Probes) conjugated to either Alexafluor 488 or 647. The cells were viewed under CFLSM. Tumor vasculature and pericyte coverage were visualized in 8- and 30-μm-thick cryosections using CD-31 (1:50 BD Bioscience) and α-SMA (1:500, Sigma) antibodies, respectively, according to established protocols [29, 30]. For studies involving immunohistochemistry, formalin-fixed paraffin-embedded (FFPE) sections of tumors were incubated with HIF-1α antibody (1:50, Sigma) and processed according to previously described protocol [31].

### Zymography

Supernatants collected from rat aortic ring wells were run on 7.5 % gel made with 0.1 % gelatin concentration. The supernatant was mixed with reducing agent-free loading dye. Electrophoresis was carried out until tracking dye was rundown, and gel was washed in washing buffer and kept overnight in reconstitution buffer in a CO_2_ incubator. It was stained with Coomassie brilliant blue dye for 20 min and then was destained with 7 % glacial acetic acid. The zymogram was scanned using a GEL DOC (Syngene).

### Statistical analysis

Data were presented as mean ± SEM. The differences between different treatments were analyzed, using the two-sided Student’s *t* test. *P* values lower than 0.05 were considered significant.

## Results

### AECHL-1-inhibited proliferation of HUVECs without hindering survival

**Fig. 1:**
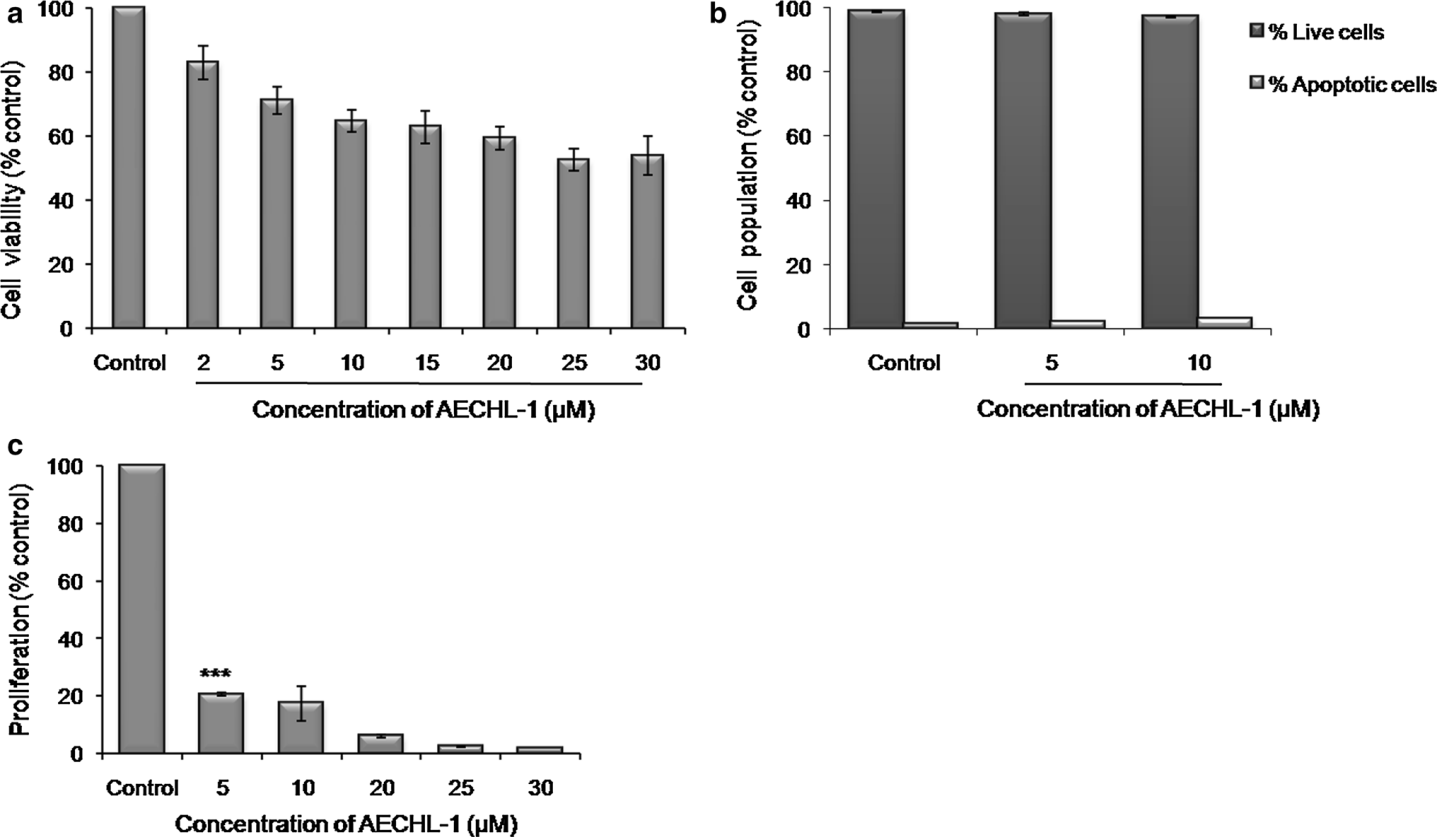
Effect of AECHL-1 on survival and proliferation of HUVECs. **a** HUVECs treated with varying concentrations of AECHL-1 for 48 h, and cell viability was determined by MTT assay. **b** HUVECS treated with varying concentrations of AECHL-1 for 24 h and subjected to flow cytometry using Annexin V-FITC as a marker of apoptosis. **c** HUVECs treated with varying concentrations of AECHL-1 for 48 h, and cell proliferation was determined by tritiated thymidine assay. Columns, mean from three independent experiments performed in triplicate; *bars*, SE. *P < 0.05, ***P < 0.001 versus control alone

HUVECS were subjected to different concentrations of AECHL-1 (0–30 μM) for 48 h, and cell viability was determined by MTT assay. With more than 65 % cells remaining viable after 48 h, 2–10 μM of AECHL-1 showed no cytotoxicity. Further increase in AECHL-1 concentration resulted in decreased viability, but only up to 60 % for 30 μM AECHL-1 (Fig. 1a). The presence of apoptotic tendency in HUVECs after AECHL-1 treatment was determined by quantifying the exposure of phosphatidyl serine (PS) on the cell membrane by flow cytometry using Annexin V-FITC assay. There was no increase seen in the % of apoptotic cells after AECHL-1 treatment. However, cell proliferation was seen to be inhibited significantly after being incubated with varying concentrations of AECHL-1 for 48 h as seen by tritiated thymidine incorporation assay (Fig. 1b). AECHL-1 5 μM was observed to be responsible for decreasing the percentage of proliferating cells by nearly 80 % (Fig. 1c). Noncytotoxic yet anti-proliferative concentrations of 5–10 μM AECHL-1 were taken for further studies. Results suggest that the apparent decrease in survival as seen by MTT is due to the anti-proliferative effect of AECHL-1 and not apoptosis.

### AECHL-1 inhibits VEGF-induced capillary structure formation, migration and invasion by endothelial cells

**Fig. 2:**
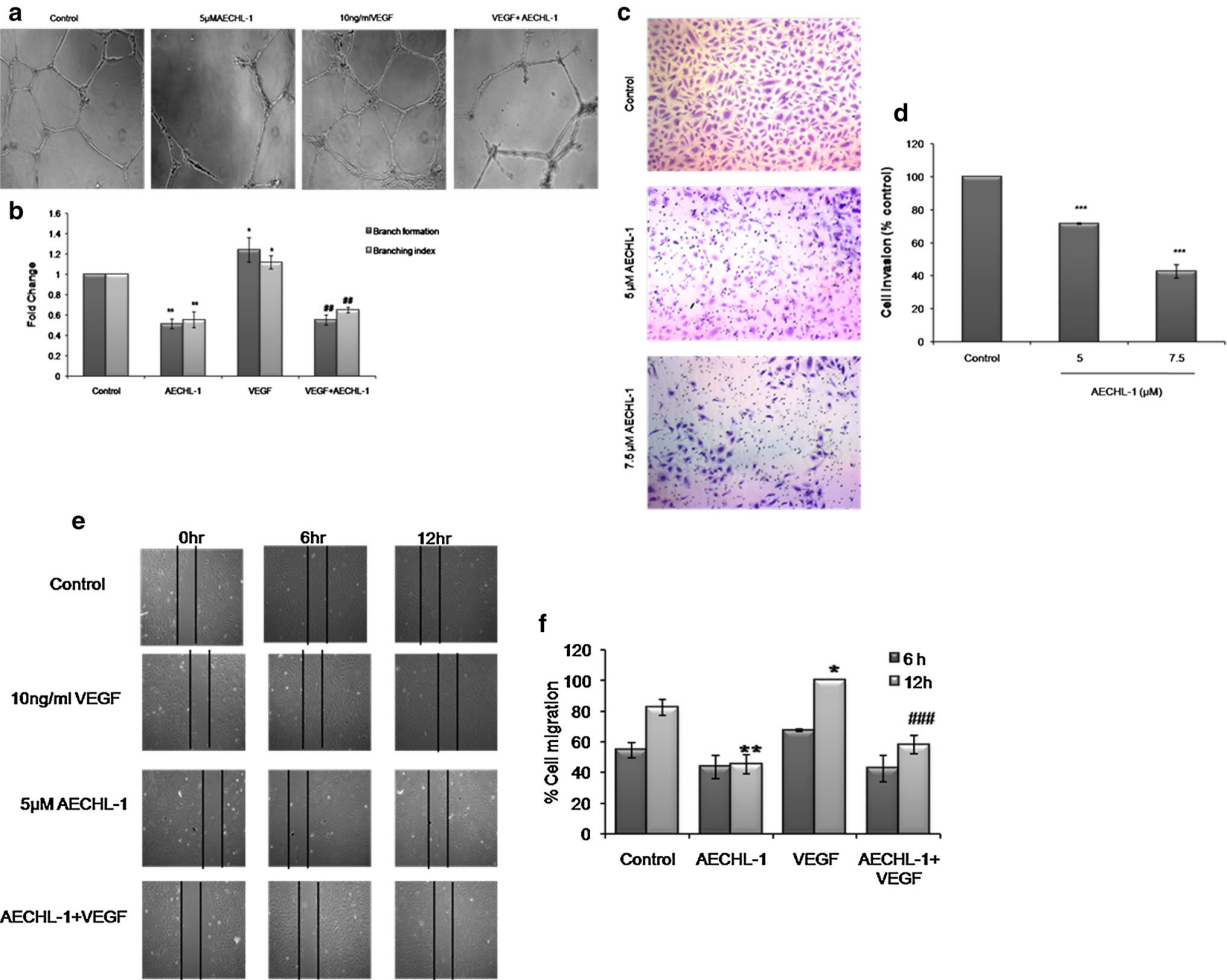
AECHL-1 inhibits VEGF-induced capillary structure formation, invasion and migration of endothelial cells. **a** AECHL-1 inhibited VEGF-induced tube formation of endothelial cells on Matrigel. **b** AECHL-1 inhibited HUVEC invasion. Migrated cells through the membrane were quantified. **c** AECHL-1 inhibited HUVEC migration. HUVEC monolayer was scratched by pipette and treated with or without 10 ng/mL VEGF. Migration is expressed as % gap closure of VEGF-treated well. Endothelial cells were photographed (magnification, ×10) using Image pro-plus and quantified using ImageJ software for all above-described experiments. Columns, mean from three independent experiments; bars, SE. *P < 0.05; **P < 0.01; ***P < 0.001 versus control and ^##^ *P* < 0.01; ^###^ *P* < 0.001 versus VEGF

As clearly illustrated in the review by Goodwin [32], morphogenesis or the formation of capillary-like structures in a polymerized 3D Matrigel matrix is an indispensable step for the formation of neo-vasculature. AECHL-1 at 5 μM could clearly inhibit capillary tube formation both independently and when stimulated with VEGF (Fig. 2a). Incomplete mesh formation was observed in wells treated with AECHL-1. Quantitative analysis revealed a decrease in both the branch length and the number of branches arising from a single node as indicated by the branch formation and branching index data, respectively (Fig. 2b).VEGF stimulation caused an expected increase in these aforementioned characteristics.

The ability of this compound in inhibiting HUVEC’s migratory and invasive potential indicated that AECHL-1 could be targeting the cytoskeleton of active endothelial cells. The percentage of actively migrating cells were 20 % higher under VEGF stimulation as compared to unstimulated or untreated control cells. AECHL-1 could, however, bring down cell migration by 60, and 40 % on receiving VEGF stimulation (Fig. 2e, f). Cell invasion assay revealed that compared to control, only 72.5 and 36.3 % cells could invade to the lower chamber in response to serum stimulation after 5 and 7.5 μM AECHL-1 treatment, respectively (Fig. 2c, d).

### AECHL-1 performs vascular pruning in the highly angiogenic chorioallantoic membrane of chick embryos

**Fig. 3:**
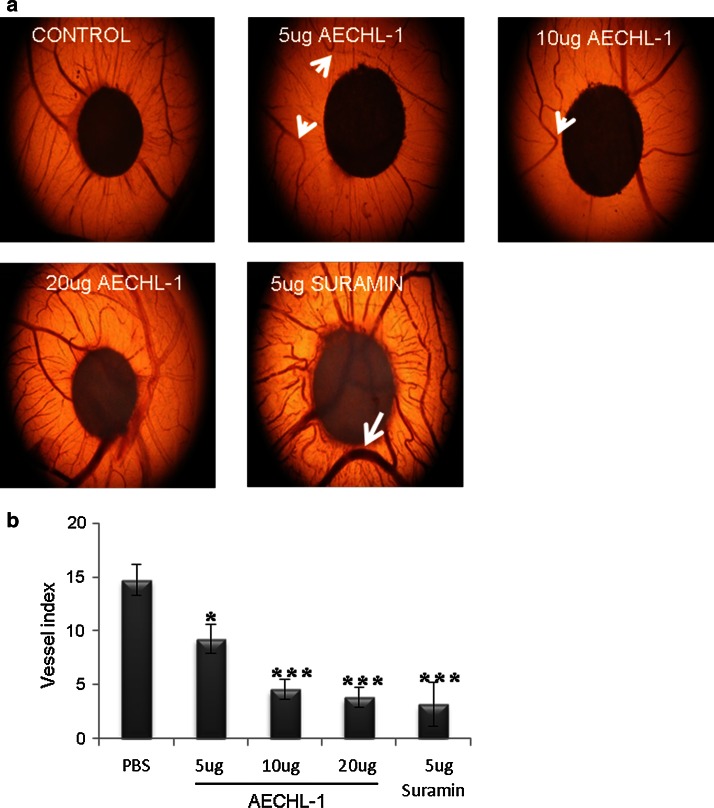
AECHL-1 performs vascular pruning in chorioallantoic membrane. **a** PBS was used for control and Suramin (5 µg) as a positive control. Tertiary vessels turn away from the site of application (*small white arrow*). Primary vessel turns away from site of application (*white arrow*).**b** No. of tertiary and quaternary vessels were quantified by manually counting three different areas from each egg per set. Columns, mean from three different experiments with *n* ≥ 5 per group; *bars*, SE. *P < 0.05; ***P < 0.05 versus control

Tumor angiogenesis causes excessive and aberrant microvessel branching as well as growth [8]. The ability to prune and normalize this abnormal vasculature is a highly desired quality in any anti-angiogenic treatment of interest [10, 33]. The anti-angiogenic effect of AECHL-1 was tested by the conventional CAM assay [34], and it was observed that all concentrations (5, 10, 20 µg) of AECHL-1 could bring about a significant decrease in tertiary and quaternary vessels, which usually form after day 6. AECHL-1 of concentrations 5 and 10 µg showed vessels clearly growing away from the site of application (Fig. 3a). None of these concentrations seemed to affect the primary or secondary vasculature present prior to receiving treatment. AECHL-1 20 µg and Suramin 5 µg successfully disrupted the vasculature but increased the tortuosity of the microvessels along with affecting the primary vasculature. Filter papers saturated with PBS were used for control eggs. Branch index: The number of tertiary and quaternary branches per egg was used as a measure of neo-vessel formation (Fig. 3b).

### AECHL-1 hinders VEGF-induced microvessel sprouting and extracellular matrix degradation, ex vivo

**Fig. 4:**
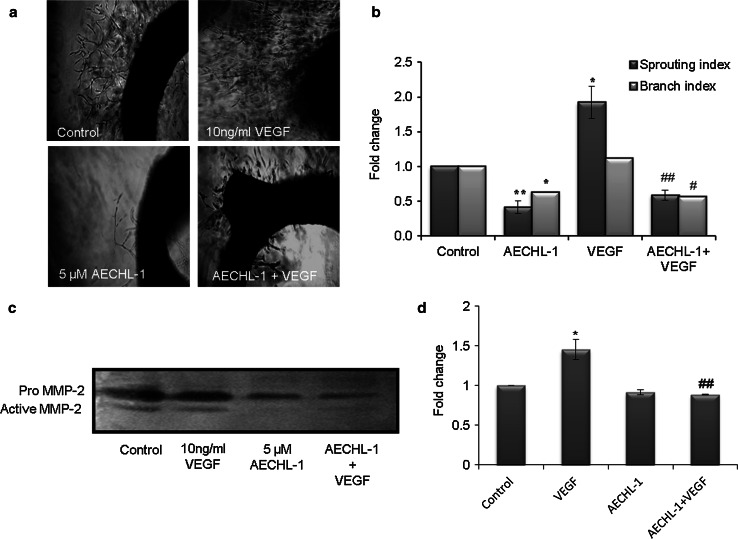
AECHL-1 inhibits VEGF-induced microvessel sprouting ex vivo. Aortic segments isolated from Wistar rats were placed in the Matrigel-covered wells and treated with VEGF in the presence or absence of AECHL-1. **a** Representative images of sprouts from the margins of aortic rings. Images were captured and quantified using Image pro-plus and ImageJ software, respectively. **b** MMP-2 activity from supernatants assayed using gelatin zymography. Quantification of band intensities was carried out using ImageJ. Columns, mean from three independent experiments; *bars*, SE. *P < 0.05; **P < 0.01 versus control and ^#^ *P* < 0.05; ^##^ *P* < 0.01 versus VEGF

A different line of evidence showing the potential of AECHL-1 in inhibiting overall angiogenesis is provided by the ex vivo model of the rat aortic ring assay [35]. Figure 4a shows that under control conditions the aortic ring was able to generate neo-vessel sprouting and that the density of these sproutings increased in the presence of the pro-angiogenic agent VEGF (10 ng/ml). AECHL-1 5 µM alone and in combination with VEGF inhibited endothelial cell sprouting. Quantification of microvessel density per treatment was depicted by their sprouting and branching index (Fig. 4b), where the former refers to the number of sprouts per ring and the latter is a measure of the number of branches per sprout.

The mechanism employed by endothelial cells for sprouting and forming neo-vasculature when stimulated involves the cleaving of extracellular matrix already surrounding the mature vessels. MMP-2 is one such matrix metalloproteinase known for actively participating in these activities. Gelatin zymography for conditioned medium from the wells containing the aortic rings subjected to AECHL-1 treatment in the presence of VEGF, revealed a decrease in MMP-2 activity as compared with the wells containing aortic rings stimulated by VEGF alone (Fig. 4c, d). AECHL-1 alone, however, could not bring any significant decrease in MMP-2 activity.

### AECHL-1 discourages tumor cell conditioned medium-induced angiogenesis in vivo

**Fig. 5:**
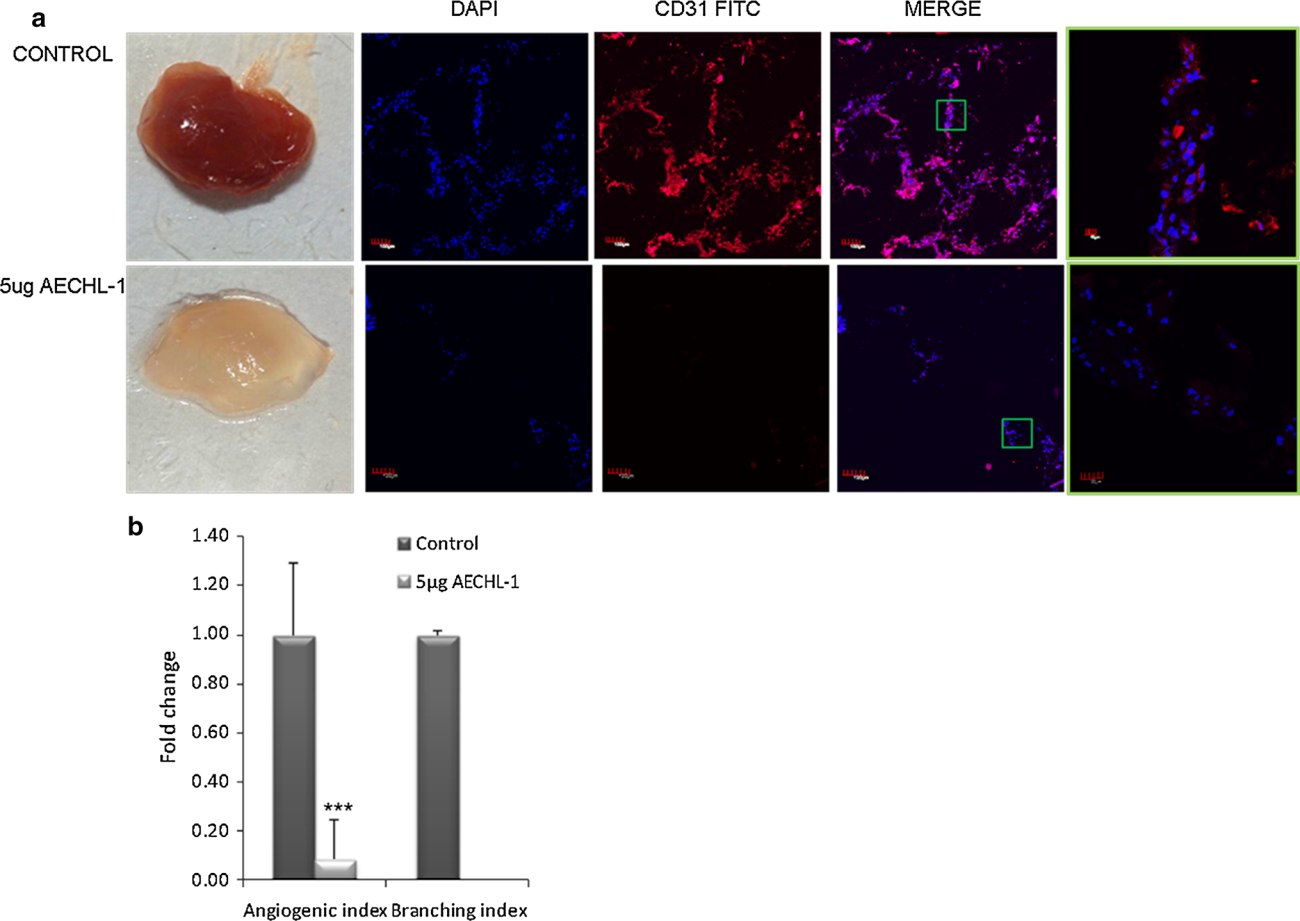
AECHL-1 inhibits MCF-7 conditioned medium-induced angiogenesis in vivo. Female SCID mice injected with 0.5 ml of Matrigel containing conditioned medium and VTH mixture. Mice were given i.p injections of AECHL-1 at 5 µg/kg of body weight. After 7 days, mice were killed and Matrigel plugs were excised. **a** Representative Matrigel plugs that were given PBS injections (control) or AECHL-1. Plugs were frozen in OCT compound and sectioned by a cryotome. Sections were subjected to CD31 FITC staining immunostaining (*pseudo-colored red*) and counterstained with DAPI. Images were taken at ×10 and ×60 magnification. **b** Quantification was carried out by ImageJ. Columns, mean from five mice per group; bars, SE. ***P < 0.001 versus control

To determine whether AECHL-1 is capable of blocking tumor-induced angiogenesis in vivo, an established in vivo angiogenesis model, the mouse Matrigel plug assay, was performed. Matrigel containing conditioned medium from MCF-7 cells was mixed with VTH mixture and subcutaneously injected into female SCID mice. Mice were given either PBS or 5 µg/kg body weight AECHL-1, i.p. Seven days later, the Matrigel plugs were excised and were photographed. Plugs from mice treated with only PBS appeared dark red in color. In contrast, plugs from mice treated with AECHL-1 were pale in color, indicating no or less blood vessel formation (Fig. 5a). Plugs were frozen in OCT compound, and their cryosections were subjected to CD 31-FITC immunostaining. Images were quantified for vessel formation; angiogenic index and branching index were used as indicators for branch length and the number of nodes (Fig. 5 b). Due to almost negligible invasion of endothelial cells into the plugs in mice treated with AECHL-1, no nodes could be quantified. In vitro studies involving conditioned media derived from AECHL-1-treated breast cancer cells also discouraged endothelial cell tube formation (see Supplementary Fig. 1a, b) and invasion (see Supplementary Fig. 1c, d). These results indicate that AECHL-1 is capable of inhibiting breast cancer cell conditioned medium-induced neo-vessel formation in vivo.

### AECHL-1 downregulates phosphorylation of VEGFR2 and downstream MAPK1/2 activity

**Fig. 6:**
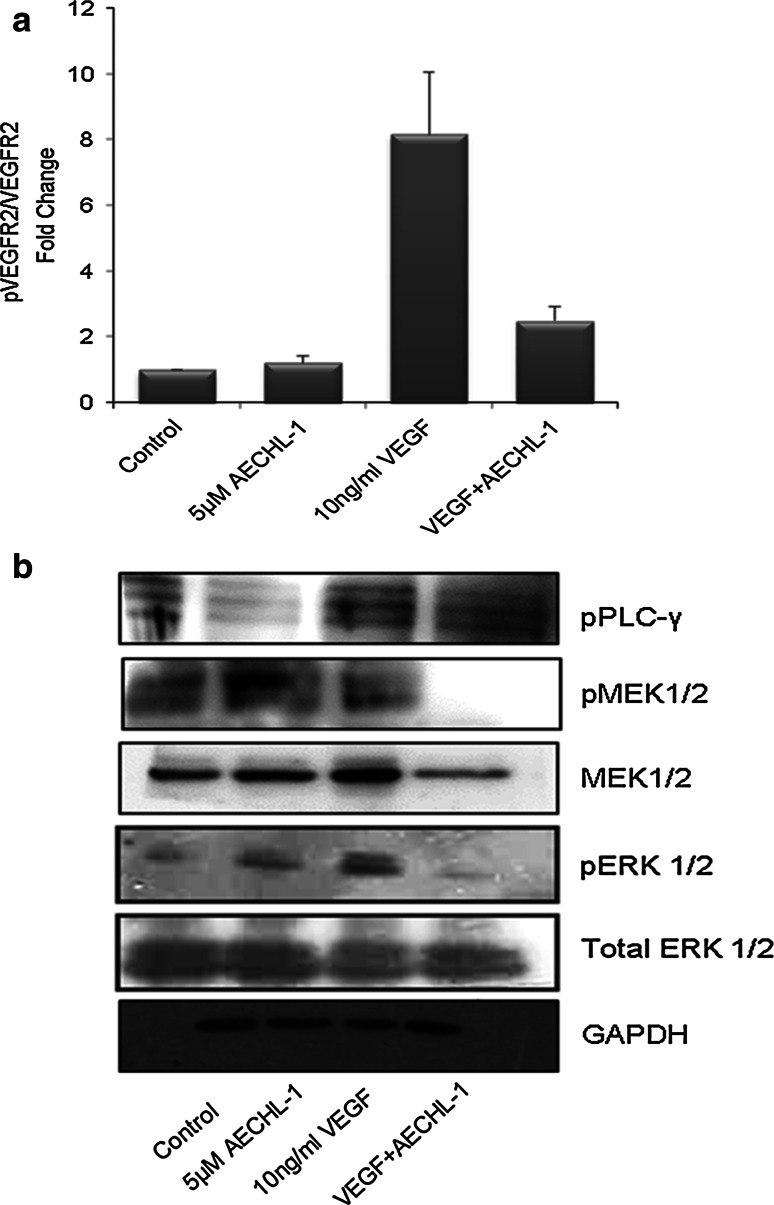
Effect of AECHL-1 on VEGFR2-mediated MAPK signaling. **a** Quantification of VEGFR2 phosphorylation by ELISA. *Fold change* indicates the ratio of phosphorylated to nonphosphorylated VEGF. Cells were pretreated with AECHL-1 for 4 h and stimulated with VEGF for 5 min. **b** Cells were pretreated with AECHL-1 for 4 h and stimulated with VEGF for 25 min. Cells were harvested, and Western blotting was performed for detection of pPLC-γ1, pMEK1/2 and pERK1/2. Membrane was stripped and reprobed with Total MEK1/2, ERK1/2 and GAPDH to indicate equal loading. Columns, mean from three different experiments performed in triplicate; *bars*, SE

To understand the mechanism by which AECHL-1 could affect endothelial cell functions involved in angiogenesis, we studied the effect of AECHL-1 on VEGFR2-tyr-1175 phosphorylation in HUVECs by ELISA. Depending on the site of phosphorylation, VEGFR2 activates various survival-, proliferation- or migration-related pathways, as a consequence deciding endothelial cell fate [36]. VEGF-mediated VEGFR2 phosphorylation (tyr-1175) was inhibited considerably by 5 μM AECHL-1 (Fig. 6a). Downstream of VEGFR-2 activation, among the major pathways responsible for cell proliferation and migration, phospholipase-C (PLC-γ1) is recruited to the membrane where it is also phosphorylated which in turn activates the MEK1/2-ERK1/2 signaling cascade [36, 37]. This pathway could be inhibited by pretreatment of HUVECs with AECHL-1, prior to VEGF induction. Interestingly, AECHL-1 alone did not seem to affect MAPK1/2 activation although it could suppress VEGFR2 phosphorylation (Fig. 6b). Hence, the anti-angiogenic activity of AECHL-1 could partly be attributed to the suppression of VEGFR2 phosphorylation upon VEGF stimulation.

### AECHL-1 suppresses cell migration by deregulating actin cytoskeletal dynamics involving downregulation of certain motility-associated proteins

**Fig. 7:**
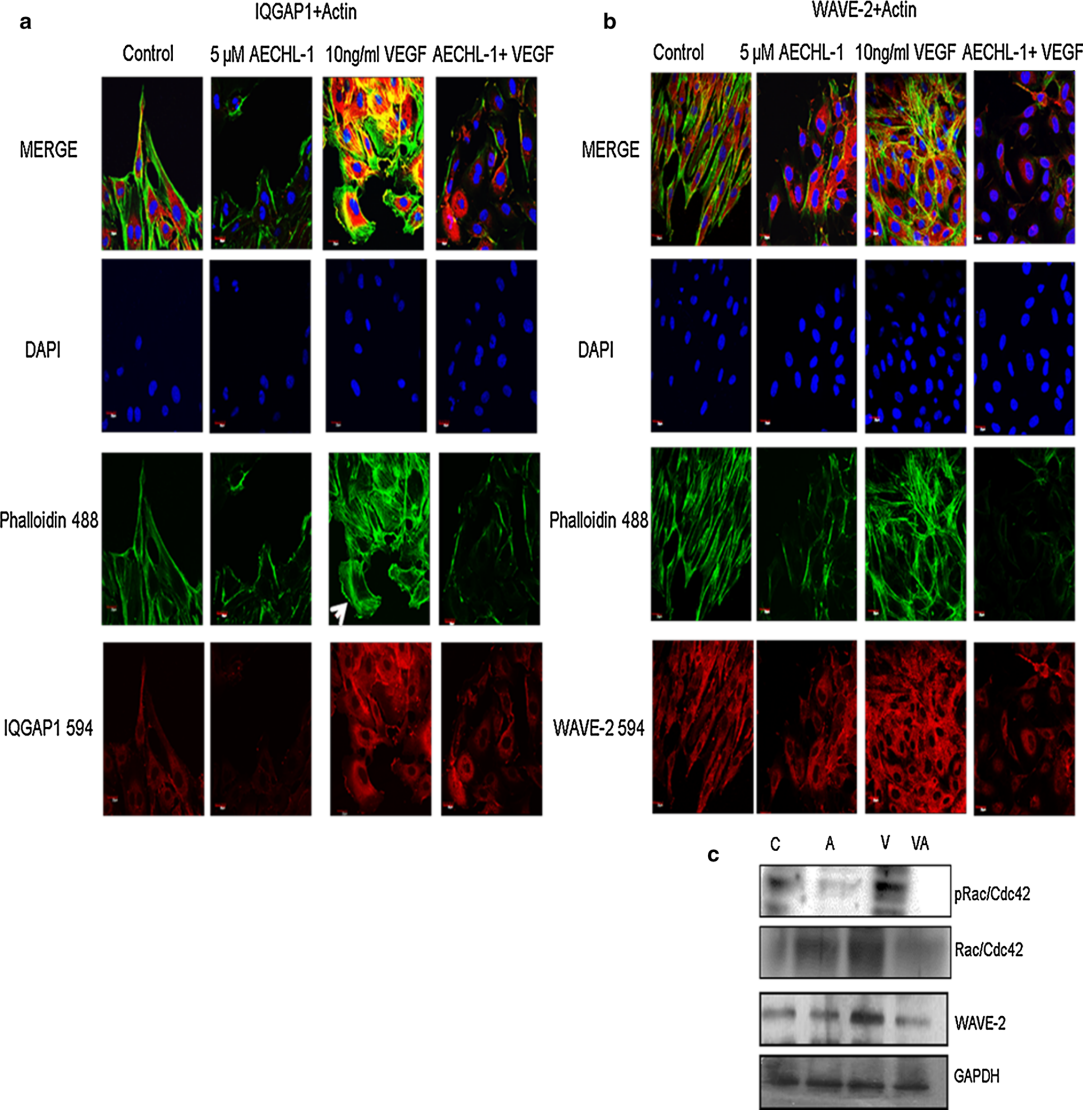
Effect of AECHL-1 on microfilament dynamics and motility-associated proteins. HUVECs were grown on coverslips till confluency and scratched with a pipette tip. Cells were pretreated with AECHL-1 for 4 h and then stimulated with VEGF and incubated for 9 h. Coverslips were then fixed and processed for immunostaining. Immunofluorescence analysis of HUVECs stained with phalloidin conjugated with Alexa Fluor 488 and **a** anti-IQGAP1 antibody **b** anti-WAVE-2 antibody. Membrane ruffles indicate active lamellipodial edge (*white arrow*). Images were taken by a confocal microscope at ×60 magnification. **c** Also at the indicated time (9 h), cells were harvested and subjected to Western blotting for detection of WAVE-2 and pRac/Cdc42. Membrane was stripped and reprobed with total Rac/Cdc42 and GAPDH to indicate equal loading

To understand the effect of AECHL-1 on actin cytoskeleton and cell motility, wound healing assay was performed on coverslips and then incubated with appropriate antibodies (phalloidin-488 or anti-WAVE-2 or IQGAP1 antibodies). It was seen that untreated cells and cells stimulated with VEGF showed increased phalloidin intensity (Fig. 7a, b), as compared to cells treated with only 5 µM AECHL-1 or both. Since phalloidin selectively binds with polymerized forms of actin, F-actin assembly could be clearly visualized in the former group. Disrupted actin assembly with the presence of only cortical actin and disappearance of stress fibers was observed in cells treated with AECHL-1 irrespective of whether they were receiving VEGF stimulus or not, although cells treated with AECHL-1 only did show the presence of a weak leading edge and higher F-actin formation. These cells were also analyzed for the presence and localization of two important proteins involved in endothelial cell motility, viz IQGAP1 [38] (Fig. 7a) and WAVE-2 [39] (Fig. 7b). IQGAP-1 was observed to be present at the leading edges localizing specifically in either filopodial extensions or active lamellipodial ruffles in control and VEGF-stimulated cells, respectively. But due to the abnormal morphology and loss of filopodial or lamellipodial extensions in the AECHL-1-treated groups, IQGAP1 could be visualized only in the cytoplasm albeit at lower intensities. WAVE-2, a Wiskott–Aldrich syndrome family verpolin homology protein, followed a similar pattern of localization, limiting itself to the cytoplasm in the presence of AECHL-1. WAVE-2 appeared to be downregulated by AECHL-1 only upon VEGF stimulation, as observed through Western blots (Fig. 7c). Since the Rac1/Cdc42 is responsible for the activation of the WAVE-2 complex [39] and hence further actin nucleation, leading to capillary lumen formation, we determined the status of Rac/Cdc42 upon VEGF stimulation and AECHL-1 treatment [40]. Also, as Rac1 is activated downstream of VEGFR-2 phosphorylation through the MAPK pathway, we saw an expected decrease in both phosphorylated and total Rac levels when ECs were pretreated with AECHL-1 followed by VEGF stimulation [36]. Thus, cells treated with both the effectors fared worse as compared to cells treated with only AECHL-1. These results echoed our previous observation that external stimulation was necessary for AECHL-1-induced inhibition of migration.

### AECHL-1 regulates HIF-1α expression and translocation both in vivo and in vitro, leading to a decrease in VEGF secretion in vitro

**Fig. 8:**
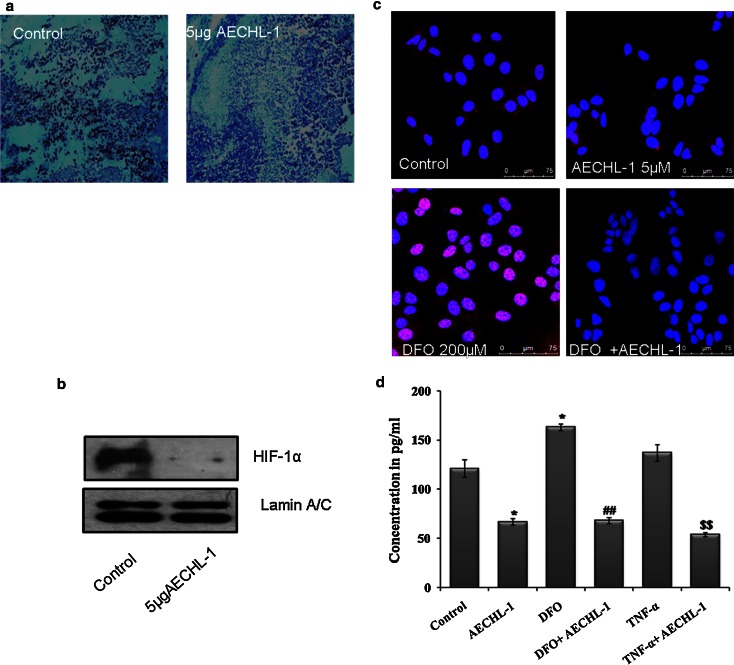
AECHL-1 regulates HIF-1α expression and translocation both in vivo and in vitro, leading to a decrease in VEGF secretion in vitro. MCF-7 cells were injected in the right flank of SCID mice(*n* = 5 per group) as described in “Materials and methods.” **a** Tumor tissues were fixed in formalin and embedded in paraffin for immunohistochemistry using anti-HIF-1α antibody. **b** Western blotting using nuclear extracts from tumor tissue revealed a decrease in HIF-1α expression. **c** AECHL-1 treatment of MCF-7 cells decreased HIF-1α stabilization and nuclear translocation as indicated by immunofluorescence studies (×60). **d** AECHL-1 treatment resulted in decreased secretion of VEGF, as determined by ELISA. TNF-α (100 µM) was used as a positive control. Columns, mean from three different experiments; bars, SE. *P < 0.05 versus control; ^##^ *P* < 0.01 versus Deferoxamine (200 µM) and ^$$^ *P* < 0.01 versus TNF-α

Tumors are essentially hypoxic due to the lack of oxygenation at the core making them more aggressive, angiogenic and metastatic, which leads to the release of pro-angiogenic cytokines and growth factor-like VEGF, among many others [41]. Hence, the tumor microenvironment plays an important role in regulating the angiogenic switch. AECHL-1 could definitively decrease VEGF secretion into the media by MCF-7 cells in the presence and absence of DFO (Fig. 8d). DFO acts as HIF-1α stabilizer ensuring increased secretion of VEGF into the media and mimicking hypoxia [42]. Images obtained from confocal microscopy-based IF analysis showed that HIF-1α nuclear translocation could be hindered when MCF-7 cells were subjected to AECHL-1 treatment, even after DFO induction (Fig. 8c). In vitro studies involving xenograft tumors were analyzed by both IHC and Western blotting. IHC analysis for HIF-1α showed a notable decrease in HIF-1α staining (Fig. 8a), when mice were treated with AECHL-1. Nuclear fractions subjected to Western blotting (Fig. 8b) confirmed that AECHL-1 could indeed downregulate HIF-1α expression and translocation in vitro and in vivo.

### AECHL-1 prunes tumor vasculature and increases mural cell involvement in an in vivo xenograft model

**Fig. 9:**
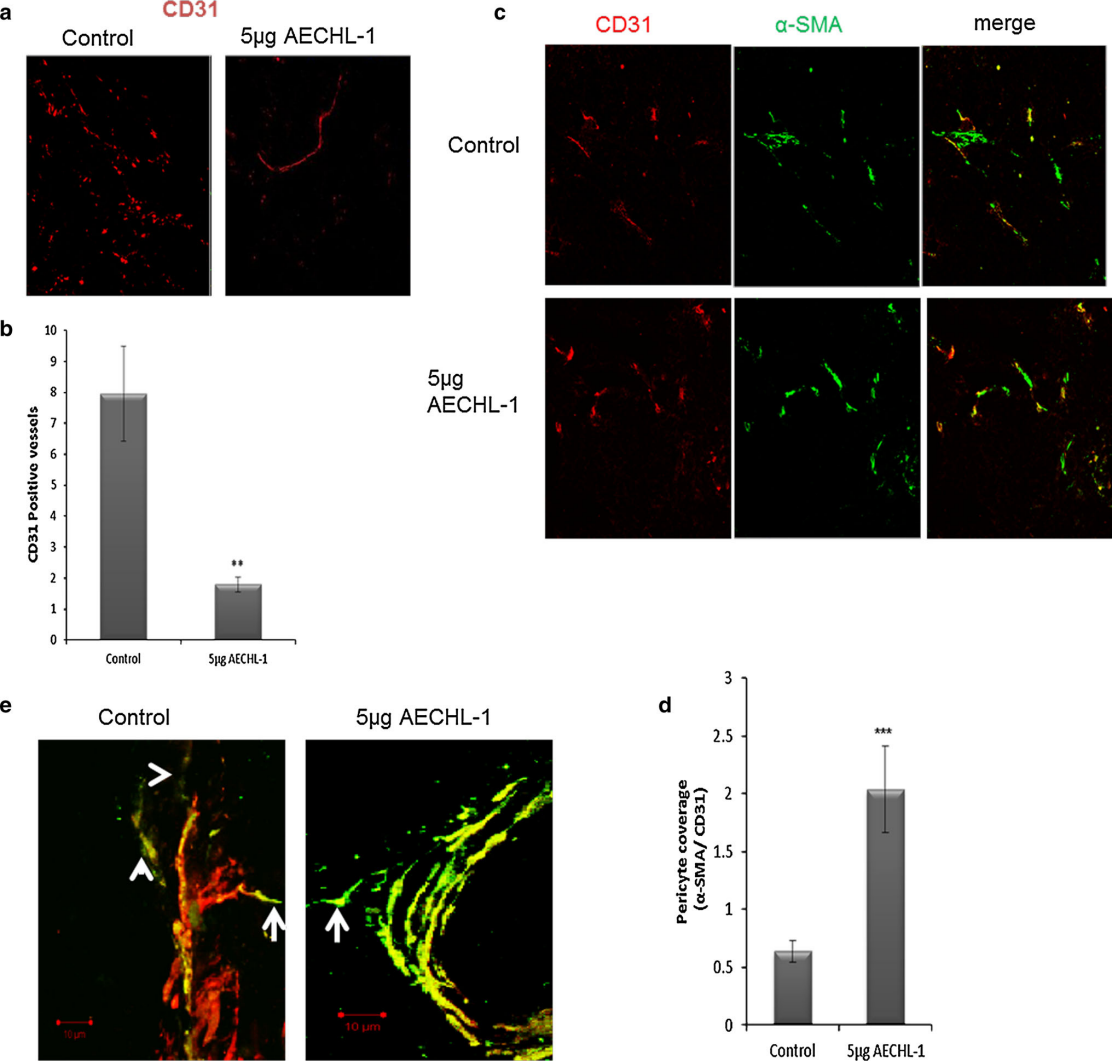
Effect of AECHL-1 on tumor angiogenesis. MCF-7 cells were injected in the right flank of SCID mice (*n* = 5 per group) as described in “Materials and methods.” Eight-micrometer cryosections were stained for CD-31 to **a** visualize and **b** quantify vessel density (×20), or CD-31 and α-SMA to **c** visualize and **d** quantify pericyte coverage of tumor vasculature. **e** Organization of mural cells in AECHL-1-treated and control tumors as analyzed in the 30-µm cryosections doublestained for CD-31 and α-SMA (×40, projected stacks).Control vessels show decreased mural cell support with treated vessels showing higher mural cell investment. *Arrows*, SMA^+^ cells extending away from vessel. *Arrowheads*, SMA^+^ cells without any associated vessels. Columns, mean from five mice per group; *bars*, SE. **P < 0.01; ***P < 0.001 versus control

Tumor vasculature is characterized by its tortuous morphology, lack of pericyte coverage and leakiness adding to ineffective oxygenation and failure to deliver chemotherapeutics [7–10, 29, 30]. Tumors from mice treated with AECHL-1 had a lower vessel density but relatively mature vasculature with significantly greater pericyte coverage as seen in the 8-μm-thick cryosections (Fig. 9a–c). Analysis of images (Fig. 9d) spanning through approximately 25–30 μm tissue at intervals of 1 μm revealed the degree of pericyte involvement with the tumor vasculature in both the treated and control groups. Tumors from control mice revealed extensively branched vascular processes with SMA-positive mural cells either extending away from the vessel with slight or no attachment, whereas tumors from the treated set had vasculature with regular morphology and an even layer of mural cells. The ability of AECHL-1 in pruning aberrantly sprouting vasculature and stabilizing the remaining ones could play an integral role in aiding tumor regression and keeping metastasis in check.

## Discussion

We have previously demonstrated that AECHL-1 displayed effective anticancer activity in numerous breast cancer cell lines among which MCF-7, a breast adenocarcinoma cell line, succumbed most effectively. AECHL-1 was also reported to have caused microtubule disruption, arrest cell cycle and induce apoptosis in tumor cells. In vivo histopathological studies suggested that AECHL-1 was selective and hence less toxic, but also effective as compared to paclitaxel and cisplatin. Histological examination of tumor tissue from nude mice showed loss of neo-vascularization and absence of hemorrhagic areas at 5 μg dose [22]. These results encouraged us to further investigate whether AECHL-1 could serve as a novel anti-angiogenic agent at subcytotoxic doses.

Our studies revealed that AECHL-1 is a highly effective anti-angiogenic agent as demonstrated in both in vitro and in vivo conditions. Proliferation, migration, morphogenesis and the ECM degradation all, indispensible stages for neo-vessel formation by endothelial cells, were checked by AECHL-1. Interestingly, all of this could be inhibited at a dose below cytotoxic levels, despite VEGF stimulation. AECHL-1 also abrogated bFGF (see supplementary Fig. 2a, b) induced tube formation and conditioned medium-induced neo-vessel formation in vitro, demonstrating AECHL-1′s ability to interfere with pro-angiogenic stimulations.

The tumor microenvironment is replete with pro-angiogenic stimulus involved in attracting and activating ECs with diverse origins and altered profiles. VEGFR2-mediated pathways are integral to stimulated EC functions; hence, we looked at the phosphorylation status of VEGFR2, when ECs are stimulated by VEGF in the presence of AECHL-1. Downregualtion of VEGFR2 phosphorylation at tyr-1175 partly explains the compound’s ability in inhibiting cell migration, invasion and proliferation through subsequent inhibition of PLC-γ1-mediated MAPK activity [36, 43, 44]. VEGF-induced phosphorylation at VEGFR2-tyr-1175 is known to bring about cell migration through the recruitment of IQGAP1 [45] at the membrane in turn activating the Rac/Cdc42 complex [46, 47]. IQGAP1 is an actin binding scaffold protein [48], which binds to Rac1 through a GAP-related domain, thus inhibiting its intrinsic GTPase activity and activating Rac1 [49–51]. As expected, IQGAP1 accumulated at the leading edge of cells stimulated with VEGF, but remained restricted to the cytoplasm in cells which were incubated with AECHL-1. This indicated the possibility that IQGAP-1-mediated Rac/Cdc 42 signaling might be targeted by AECHL-1. To further confirm the inhibition of Rac-/Cdc42-mediated signaling, we looked at both Rac1/Cdc42 activation and WAVE-2 a Wiskott-Aldrich Syndrome Protein family Verprolin—homologous proteins (WAVEs). WAVE proteins are known to act downstream of Rac/GTPase (Rho family small GTPase) [36], connecting Rac activation to the induction of Arp2/3-mediated actin polymerization [52, 53]. WAVE-2 was indeed seen to avoid cell membranes and showed a decrease in expression when incubated with AECHL-1 prior to VEGF stimulation as compared to VEGF-stimulated cells. This observation could be attributed to the inhibition of Rac1/Cdc42 activation. The fact that cells treated with only AECHL-1 did not show a decrease in either Rac or WAVE-2 levels could imply that AECHL-1 may be involved in targeting Actin cytoskeleton dynamics much more effectively in response to external stimuli. Further validation for this theory is derived from the observation that cells treated with only AECHL-1 fared better than cells receiving both the treatments. The former showing the presence of a leading edge and relatively better F-actin organization.

It is also interesting to note the difference in the pattern of actin cytoskeleton assembly in response to different treatments in these cells. Ruffled lamellipodial leading edges served the purpose of cells at the leading edge responding to VEGF stimulus better, whereas untreated cells although sporting a healthy morphology showed the presence of laterally arranged stress fibers, usually seen in cells with strong focal adhesions. This could be indicative of cells responding to only a scratch-inflicted injury on the monolayer than an external stimulus.

It has been seen that endothelial cells in tumor vasculature are comparatively more proliferative than their counterparts in mature adult vasculature and hence are susceptible to vasculature-disrupting drugs at low doses [54, 55]. Thus, it is not surprising that AECHL-1 could be exploiting this differential response phenomenon in targeting actin cytoskeletal dynamics, motility-related proteins actively proliferating stimulated endothelial cells.

Breakdown of the extracellular matrix (ECM) by Matrix Metalloproteinases encourages endothelial cell sprouting and allows for greater freedom in motility and invasion. AECHL-1 was seen to inhibit MMP-2 activity in response to VEGF stimulation in the rat aortic ring assay. This assay also allows the study of endogenous growth factors being secreted into the media by the implants and the interaction of their outgrowths with vasculature supportive cells such as pericytes and smooth muscle cells over a period of time. This provides much needed insight into the mode of action of our compound. Apparently, AECHL-1 could suppress angiogenic sprouting in aortic rings not receiving VEGF stimulus, maybe due to reduced secretion of pro-angiogenic cytokines and growth factors and the ability of AECHL-1 to interfere in events other than cytoskeletal dynamics. This observation prodded us to investigate whether AECHL-1 could downregulate pro-angiogenic growth factor expression in tumorigenic cells. We observed that VEGF secretion could be downregualted by AECHL-1 even when hypoxia-mimicking agents such as DFO or pro-angiogenic cytokines such as TNF-α [56] were administered. Hypoxia causes the stabilization of HIF-1α, which directly promotes VEGF expression by binding upstream of its promoter [57].

**Fig. 10:**
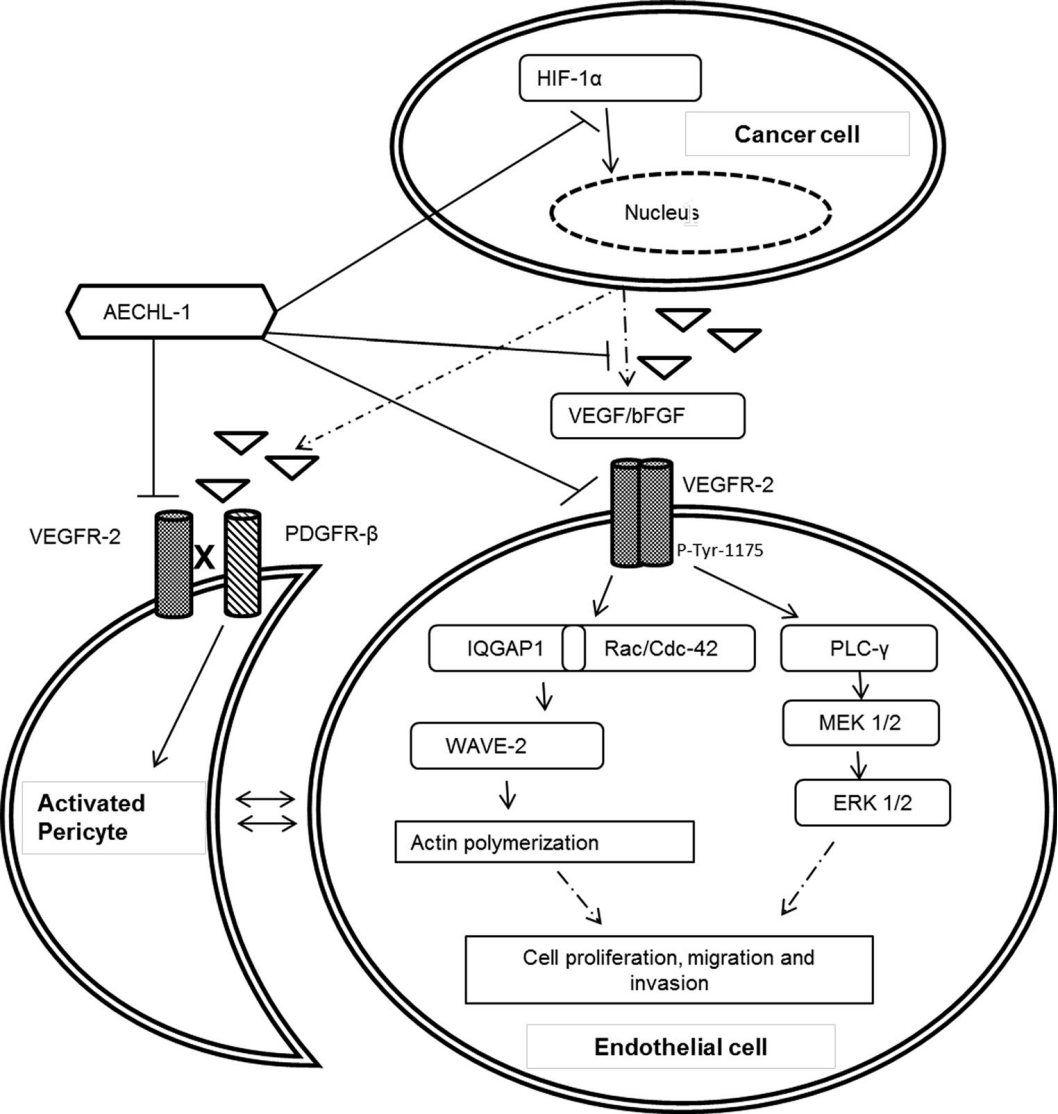
Schematic representation of the molecular mechanisms involved in regulation of endothelial cell function and tumor angiogenesis by AECHL-1

Most anti-angiogenic agents have been implicated to be countereffective by making tumors even more hypoxic and hence aggressive by destructing tumor vasculature completely [58, 59]. This also results in ineffective delivery of therapeutic agents to the tumor. The practice of vessel normalization advocates the necessity of aberrant and tortuous tumor vasculature to be pruned and normalized aiding in delivery of required therapeutics and oxygen, leading to reduced hypoxia and faster tumor regression [10, 33]. In our *in ovo* CAM assay, the primary and secondary vasculature were seen to be unaffected at 5 and 10 µg doses, but performed extensive vascular pruning of tertiary and quaternary vessel. At higher doses, AECHL-1 and Suramin treatment resulted in aberrantly disrupted vasculature along with disturbed primary vasculature making these treatments an unattractive option for further perusal. While in tumors harvested from AECHL-1-treated mice, the vessels were supported by a firm layer of pericytes indicating mature vasculature, since abnormal neo-vasculature shows a lack of pericyte involvement. Data arising out of both *in ovo* and in vivo assays bode well for a prospective therapeutic agent, as it hints toward the elimination as wells as a possibility for normalization of erratic vasculature in response to pro-angiogenic stimuli, without harming, preexisting vasculature. The decrease in HIF-1α levels in MCF-7 xenograft tumors could have a multipronged effect where it not only abates concerns about tumor aggressiveness caused by the severe hypoxic conditions generated by anti-angiogenic treatments but also increases the chances of vascular normalization by increasing pericyte coverage, since HIF-1α directly influences VEGF production and in turn antagonizes pericyte involvement with ECs by activating VEGFR2 and downregulating PDGFR-β signaling through a VEGFR2–PDGFR-β complex [60]. The extensive perivascular support observed in tumors from AECHL-1-treated mice could also be due to the decrease in HIF-1α levels. The above-described molecular mechanisms thus encompass the multiple targets of AECHL-1 involved in impairing stimulated endothelial cell function and tumor vessel normalization (Fig. 10).

In conclusion, the observation that AECHL-1 portrays an anti-angiogenic role at subcytotoxic doses, by selectively targeting aberrant and immature vasculature, can be exploited further for therapeutic purposes.

## Electronic supplementary material

Supplementary Fig. 1 AECHL-1 inhibits capillary structure formation and invasion of endothelial cells when incubated with conditioned media derived from MCF-7 cells treated with Deferoxamine (DFO, 200 µM). a,b AECHL-1 inhibited DFO-induced tube formation of endothelial cells on Matrigel. c,d AECHL-1 inhibited HUVEC invasion. Migrated cells through the membrane were quantified in the Transwell assays. After incubation, endothelial cells were photographed (magnification, × 100) using Image pro-plus and quantified using ImageJ software for above-described experiments. Columns, mean from three different experiments; bars, SE. *, P < 0.05; **, P < 0.01;*** P< 0.001 versus control and ##, P<0.01;###, P<0.001 versus DFO

Supplementary Fig. 2 AECHL-1 inhibits capillary structure formation and VEGFR2 activation in endothelial cells when stimulated with bFGF. a, b AECHL-1 inhibited 10ng/ml bFGF-induced tube formation of endothelial cells on Matrigel. After incubation, endothelial cells were photographed (magnification, × 100) using Image pro-plus and quantified using ImageJ software for above-described experiments. c Quantification of VEGFR2 phosphorylation by ELISA. Fold change indicates the ratio of phosphorylated to nonphosphorylated VEGFR2. Cells were pretreated with AECHL-1 for fourth and stimulated with bFGF for 5 mins. Columns, mean from three different experiments; bars, SE. *** P< 0.001 versus control
