# Supplementary figures and images for: AECHL-1, a novel triterpenoid, targets tumor neo-vasculature and impairs the endothelial cell cytoskeleton
Source: Angiogenesis. 2015 May 8;18(3):283–99. doi: 10.1007/s10456-015-9466-5 (PMC4472952; doi:10.1007/s10456-015-9466-5)

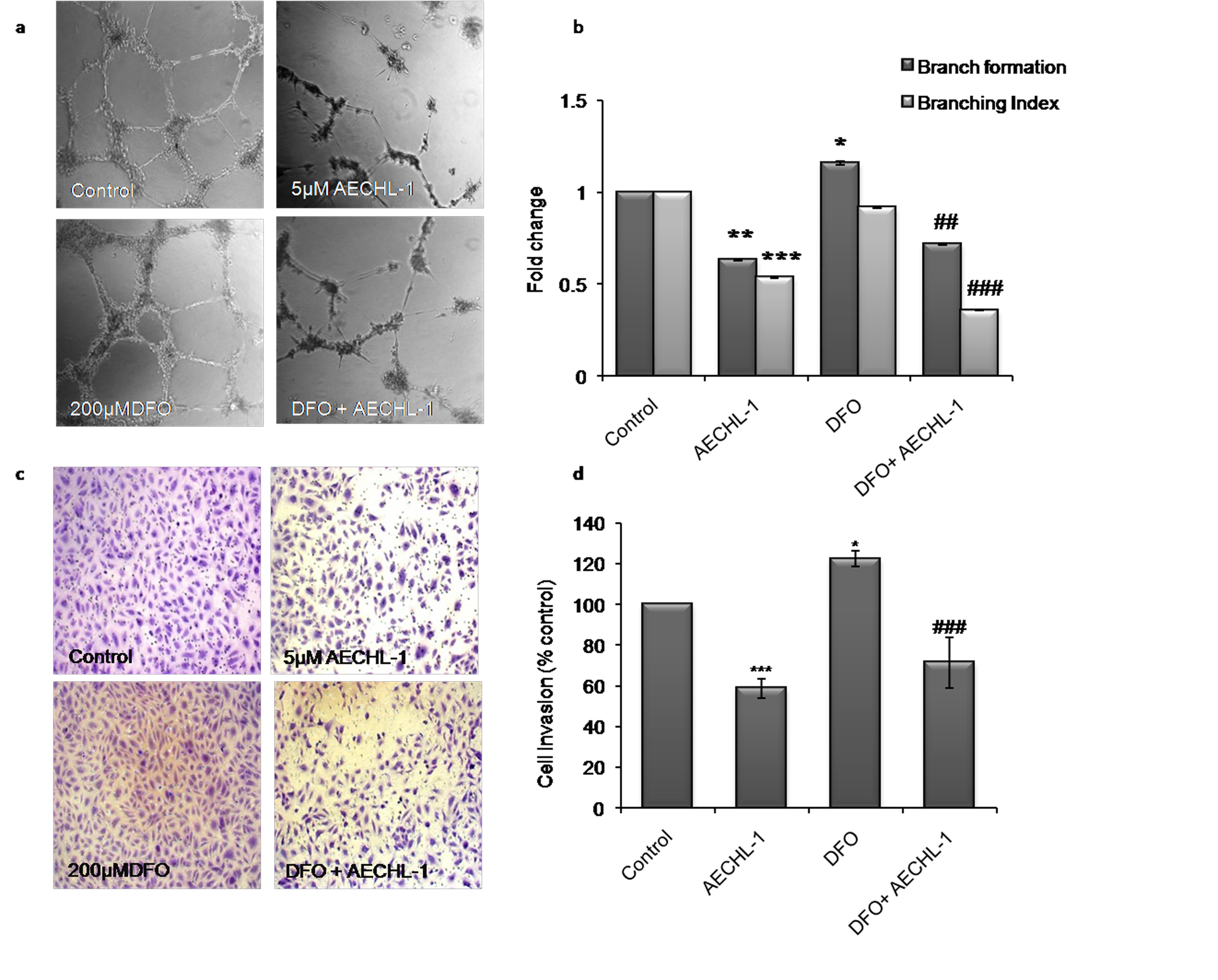

Supplement: Supplementary file 1 — AECHL-1 inhibits capillary structure formation and invasion of endothelial cells when incubated with conditioned media derived from MCF-7 cells treated with Deferoxamine (DFO, 200 µM). a,b AECHL-1 inhibited DFO-induced tube formation of endothelial cells on Matrigel. c,d AECHL-1 inhibited HUVEC invasion. Migrated cells through the membrane were quantified in the Transwell assays. After incubation, endothelial cells were photographed (magnification, × 100) using Image pro-plus and quantified using ImageJ software for above-described experiments. Columns, mean from three different experiments; bars, SE. *, P < 0.05; **, P < 0.01;*** P< 0.001 versus control and ##, P<0.01;###, P<0.001 versus DFO [file 10456_2015_9466_MOESM1_ESM.tif]

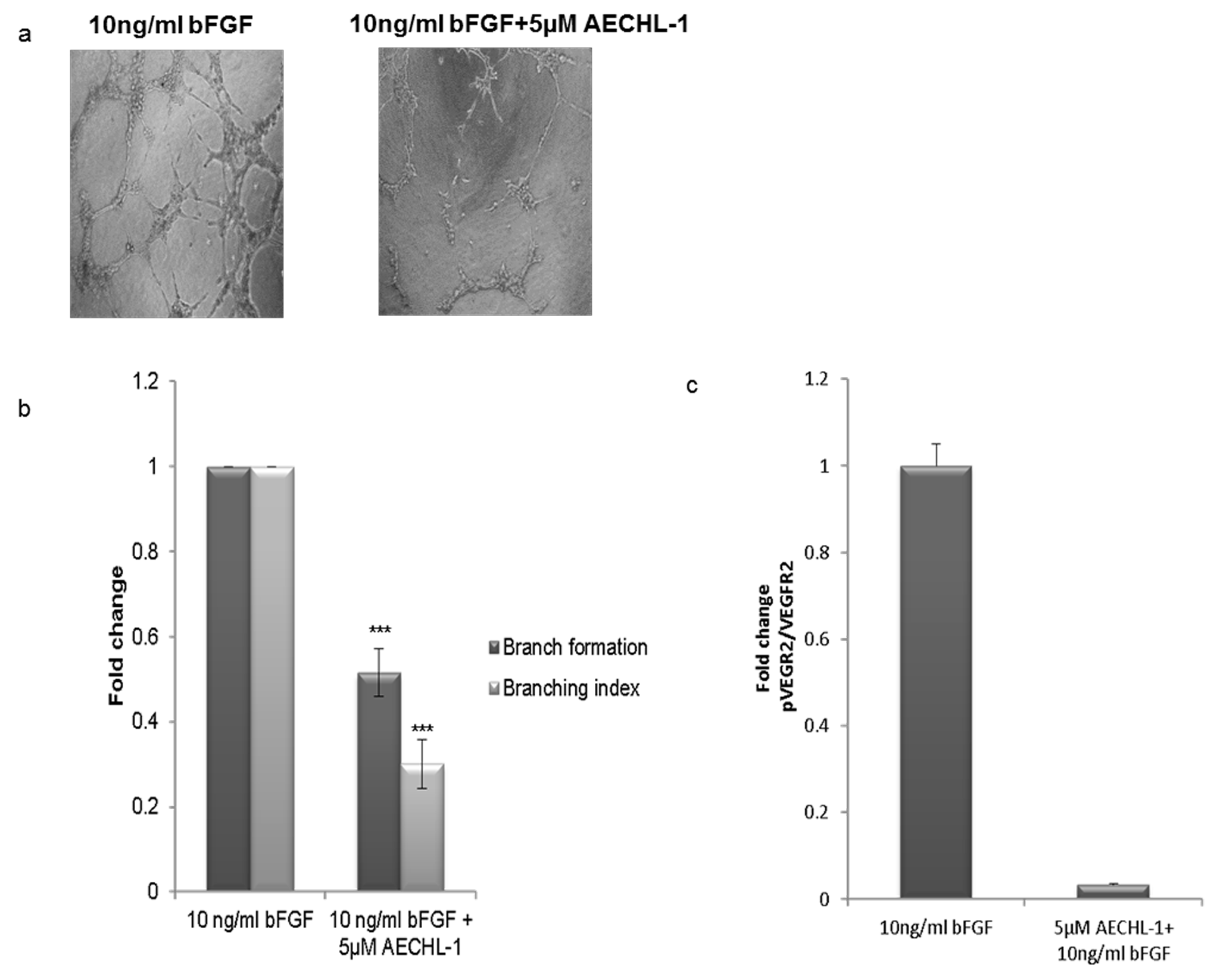

Supplement: Supplementary file 2 — AECHL-1 inhibits capillary structure formation and VEGFR2 activation in endothelial cells when stimulated with bFGF. a, b AECHL-1 inhibited 10ng/ml bFGF-induced tube formation of endothelial cells on Matrigel. After incubation, endothelial cells were photographed (magnification, × 100) using Image pro-plus and quantified using ImageJ software for above-described experiments. c Quantification of VEGFR2 phosphorylation by ELISA. Fold change indicates the ratio of phosphorylated to nonphosphorylated VEGFR2. Cells were pretreated with AECHL-1 for fourth and stimulated with bFGF for 5 mins. Columns, mean from three different experiments; bars, SE. *** P< 0.001 versus control [file 10456_2015_9466_MOESM2_ESM.tif]
